# Supplementary material for: Evidence supported by Mendelian randomization: impact on inflammatory factors in knee osteoarthritis
Source: Front Med (Lausanne). 2024 May 28;11:1382836. doi: 10.3389/fmed.2024.1382836 (PMC11165061; doi:10.3389/fmed.2024.1382836)
Supplement: Supplementary file 1 [file Data_Sheet_1.ZIP › data/GCST90274792_5e6/792.forest.pdf]

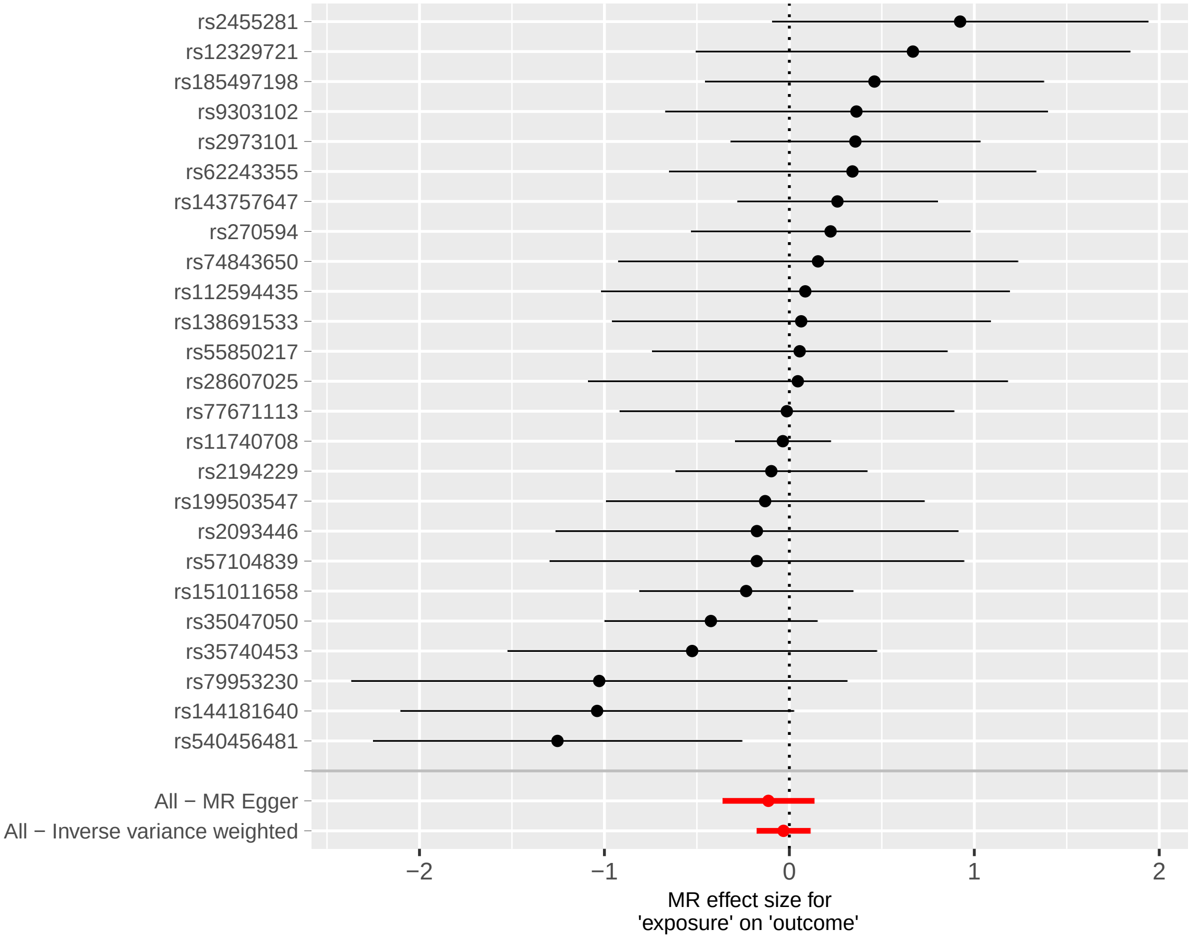

rs2455281
rs12329721
rs185497198
rs9303102
rs2973101
rs62243355
rs143757647
rs270594
rs74843650
rs112594435
rs138691533
rs55850217
rs28607025
rs77671113
rs11740708
rs2194229
rs199503547
rs2093446
rs57104839
rs151011658
rs35047050
rs35740453
rs79953230
rs144181640
rs540456481
All − MR Egger
All − Inverse variance weighted
−2
−1
0
1
2
MR effect size for
'exposure' on 'outcome'
